# Lost in clocks: non‐canonical circadian oscillation discovered in *Drosophila* cells

**DOI:** 10.15252/msb.20188567

**Published:** 2018-09-24

**Authors:** Koji L Ode, Hiroki R Ueda

**Affiliations:** ^1^ Department of Systems Pharmacology Graduate School of Medicine The University of Tokyo Tokyo Japan; ^2^ Laboratory for Synthetic Biology Center for Biosystems Dynamics Research RIKEN Osaka Japan; ^3^ International Research Center for Neurointelligence (WPI‐IRCN) UTIAS The University of Tokyo Tokyo Japan

**Keywords:** Genome-Scale & Integrative Biology, Metabolism, Quantitative Biology & Dynamical Systems

## Abstract

In most organisms, cell‐autonomous circadian clocks are driven by a transcription–translation negative feedback loop (TTFL). *Per* was the first identified clock gene in the fruit fly and is a core component of the circadian TTFL. Surprisingly, in their recent study, Rey *et al* (2018) demonstrate the presence of apparent circadian rhythmicity in a fly cell line that does not express several core clock genes including *per* (Rey *et al*, 2018). Quantitative multi‐omics measurements allowed the identification of unknown oscillating components and revealed hundreds of transcripts, proteins, and metabolites showing 24‐h rhythmicity, suggesting that at least in the fly, the circadian clock may be driven by non‐canonical (i.e., independent of *per*‐driven TTFL) molecular mechanisms.

The fruit fly *Drosophila melanogaster* has been one of the most important model organisms for the analysis and understanding of circadian clocks. The identification of *per* as the gene responsible for behavioral circadian rhythmicity led to the canonical molecular model of circadian oscillator, where PER protein inhibits its own transcription through the interaction with other transcription regulators such as TIM and CLK (Fig 1). This TTFL is further controlled by post‐translational regulation such as PER phosphorylation catalyzed by the kinase DBT (Doubletime), a homolog of mammalian Casein Kinase I (CKI). The overall structure of TTFL appears to be conserved in terms of the regulatory relationships between core circadian factors among other organisms, and the TTFL model has been considered as a canonical circadian oscillator.

**Figure 1:**
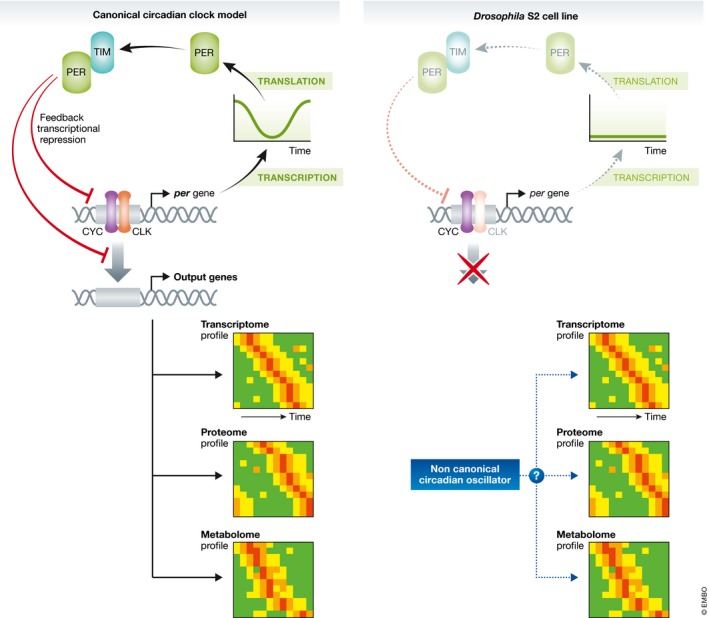
Circadian rhythmicity observed in the absence of canonical clock gene expression CYC and CLK form a heterodimer that binds to a promoter element and activates the expression of downstream genes. *per* gene expression is activated by CYC/CLK. PER protein then binds to TIM and represses the activity of CYC/CLK. The overall mechanism creates a negative feedback loop. This negative feedback loop is considered as the core circadian oscillator in *Drosophila*, generating circadian oscillation in many transcripts, proteins, and metabolites. *Drosophila* S2 cells do not express CLK, PER, and TIM; thus, the core feedback mechanism does not operate in this cell line. Surprisingly, Rey *et al* (2018) found hundreds of transcripts, proteins, and metabolites that show apparent circadian rhythmicity under constant culture conditions, suggesting that this rhythmicity is generated through non‐canonical circadian mechanisms.

However, several mechanisms driving circadian clocks are independent of the TTFL. For example, the circadian clock in cyanobacteria, driven by KaiA, KaiB, and KaiC, is an established example of a TTFL‐independent mechanism. When KaiC is incubated with KaiB, KaiC, and ATP *in vitro*, the phosphorylation status of KaiC oscillates with a period of 24 h, indicating that a post‐translational mechanism that does not involve transcription/translation is the core circadian oscillator in this prokaryotic organism (Nakajima *et al*, 2005). The non‐TTFL model was extended to eukaryotic cells by findings of circadian oscillations in the oxidation status of peroxiredoxin in enucleated mammalian red blood cells as well as nucleated cells (O'Neill & Reddy, 2011). Nonetheless, the extent to which the circadian clocks of non‐cyanobacteria organisms rely on canonical TTFL mechanisms, or on other non‐TTFL mechanisms, is still under discussion, and the implications for cellular and organism‐level circadian physiology remain unclear.

The study by Reddy and colleagues provides evidence indicating that circadian oscillations not driven by the canonical TTFL can be observed for hundreds of transcripts, proteins, and metabolites (Rey *et al*, 2018). This was demonstrated using *Drosophila* S2 cells, which do not express *per*,* tim,* and other core components of the canonical circadian TTFL. Up to now, circadian oscillations had not been detected using the classic circadian reporters that rely on the canonical TTFL model (e.g., the expression dynamics of *per*). To identify molecules showing circadian rhythmicity, Rey *et al* used quantitative omics approaches. They collected time‐course samples of S2 cells for 57 h at 3 h intervals under carefully controlled conditions and used these samples to perform RNA‐seq, mass spectrometry‐based proteomics and metabolomics analyses. Genes and proteins involved in metabolic processes were found to be enriched among the rhythmic components. In agreement with this finding, the abundance of several metabolites showed circadian rhythmicity. Interestingly, the rhythmic metabolites include ATP and NADP, which often regulate enzymatic activities as a substrate or cofactor. Such metabolic rhythmicity may not simply be the readout of protein oscillations—it is also possible that the metabolic oscillations can in turn regulate protein activities. Considering that the concentration of ATP changes ~2‐fold between peak and trough in their analysis, one can readily imagine that this non‐canonical circadian oscillation may impact many aspects of cellular physiology as well.

Rey *et al* might have discovered a new circadian clock that has remained unknown so far due to the focus on the canonical mechanisms regulating the circadian clock. Does a *de novo* autonomous oscillator exist behind the observed rhythmicity in the absence of a canonical circadian clock? If the non‐canonical oscillator has intrinsic 24 h periodicity, then S2 cells will be preferentially synchronized close to the 24 h temperature cycle, as used in this study, but not well synchronized by a cyclic signal at different frequencies. This can be tested in future studies. In addition, if the non‐canonical circadian rhythmicity is driven by an actual “circadian” oscillator, the period of oscillation is expected to be around 24 h regardless of the period of the temperature cycle used for the synchronization. In order to perform rigorous analyses of the oscillator's properties, it is important to find a robust reporter that represents the non‐canonical circadian rhythmicity. The multi‐omics dataset presented in this study is a useful resource for finding such a reporter, which will then allow genetic and chemical screens to discover components regulating 24‐h period length. This strategy has been previously employed to find core components involved in the canonical TTFL.

A potential candidate regulator could be the kinase DBT, since DBT and its mammalian homolog CKI are not only critical for period determination of the canonical TTFL‐based clock but also control the peroxiredoxin cycle period (O'Neill *et al*, 2011). Notably, a recent study demonstrated that this kinase also controls the period of (non‐circadian) metabolic oscillations in yeast (Causton *et al*, 2015). Future analyses in this direction may uncover the components driving this newly reported circadian rhythmicity and resolve whether the oscillation is founded on a TTFL, post‐translational mechanisms, metabolic networks, or their combination. Another important feature characterizing the circadian oscillation is temperature compensation. Circadian clocks do not conform to the usual biochemical principle that would mean that high temperatures shorten the period of the clock and low temperatures extend it. Instead, when temperature is kept constantly low or high, period remains invariant. Accordingly, Rey *et al* examined whether the period of S2 cell rhythmicity is affected by changing the incubation temperature. Although their results supported that period is not affected by temperature, the fact that S2 cells do not tolerate temperatures higher than 28°C limits the range of temperature change that could be tested experimentally. Further evaluation of temperature compensation over a wider range of physiological temperatures would need to be undertaken in future work.

Although the study by Rey *et al* focused on a particular *Drosophila* cell line, it is reasonable to speculate that the non‐canonical circadian oscillator operates in other *Drosophila* cells, in which the *per*‐driven canonical circadian oscillator exists. It would be interesting to examine whether the non‐canonical rhythmicity can be observed in cells in which the “canonical” clock genes have been knocked out. It is also possible that similar non‐canonical circadian rhythmicity could exist in other organisms. Interestingly, multi‐omics datasets from mammals share some features found in the multi‐omics profiles of the *Drosophila* cells with non‐canonical circadian rhythmicity. For example, a comparison between RNA‐seq and proteomics profiles from mouse liver highlighted the uncoupling of rhythmic transcripts and rhythmic proteins and the large time delay between the peak phase of transcripts and corresponding proteins (Mauvoisin *et al*, 2014; Robles *et al*, 2014). According to the canonical TTFL‐based model, such complex behavior is generated through the downstream modification of canonical circadian oscillator output, but it is possible that non‐canonical circadian mechanisms are also responsible for complex rhythmic behavior.

Identification of the underlying molecular mechanisms and evaluation of its physiological significance are important future directions that will provide more conclusive support for the presence of a non‐canonical and *de novo* circadian clock responsible for the apparent circadian rhythmicity observed in the study by Rey *et al*. If cells are equipped with multiple coupled circadian oscillators, this will change the way we think of the overall organization of circadian rhythmic output within the cell.
